# Playing music together: Exploring the impact of a classical music ensemble on adolescent’s life skills self-perception

**DOI:** 10.1371/journal.pone.0306326

**Published:** 2024-07-11

**Authors:** Anna Bussu, Marta Mangiarulo

**Affiliations:** 1 School of Law and Criminal Justice, Edge Hill University, Ormskirk, United Kingdom; 2 University of Leicester, Leicester, United Kingdom; The Open University of Israel, ISRAEL

## Abstract

This paper explored the effectiveness of ensemble performance on the development of adolescent’s life skills. An explorative qualitative study investigated young musicians’ self-perception about the benefits and challenges of learning and playing music together. A convenience sampling technique was adopted for interviewing 15 adolescents (12–18 years old) who participated in a long-term music education programme led by a charity in the North-West of England. The data were analysed using NVivo, employing a thematic analysis approach. Two main themes emerged from the analyses: (1) the main benefits of playing and learning in an ensemble: the development of music and life skills; (2) the challenges experienced by the musicians learning in the ensemble. The findings suggest that participants were conscious of the positive effects of playing in an ensemble on their lives. This extended beyond merely learning a musical instrument, i.e. acquiring music skills. In particular, young musicians recognised they had developed greater self-confidence and cognitive skills such as critical thinking and self-awareness. Primarily, they developed effective communication and interpersonal skills. At the same time, these young musicians recognised they had to face challenges related to the process of learning music in an ensemble, such as managing emotions of frustration and adapting to different music learning styles and techniques. Finally, suggestions are made for the implementation and evaluation of future projects to explore the impact and effectiveness of classical music programmes, with a particular emphasis on ensemble-based initiatives and their influence on life skills.

## Introduction

The literature on the benefits of listening to and playing music for children and young people is ever-growing. Several studies have been published on the benefits of music across the lifespan [1], including early childhood [2–4], adolescence [2, 5], and older adulthood [6].

In 2019, the World Health Organization (WHO) issued a scoping review [7] of evidence-based studies indicating that listening to, making, and playing music is associated with psycho-social wellbeing, lower levels of stress, and lower daily anxiety [7, 8]. Music-based interventions have been utilised to improve public health, reduce inequalities, and promote adolescent wellbeing. They showed a positive impact on the self-confidence and engagement of disadvantaged young people, enabling them to express themselves positively [9–11].

Moreover, music represents an important protective factor for young people’s health and wellbeing; research has provided evidence of effectiveness across physical and psychological dimensions [1, 7] and on social and mental wellbeing [12]. Listening to music [13] and playing music [14] enable young people’s self-emotional regulation and help them to connect with others.

Playing music is an important activity for developing children’s and young people’s life skills, which can be defined as essential psycho-social competencies and interpersonal skills that enable people to deal effectively with the demands and challenges of life [15]. Among the examples of transferable life skills for young people, effective communication emerges as a cornerstone, playing a pivotal role in fostering interpersonal relationships and adeptly navigating diverse social interactions. Furthermore, in light of the progressively intricate decisions encountered by youths, the cultivation of robust decision-making abilities assumes paramount importance [16, 17].

The literature on life skills encompasses a diverse array of terminologies and definitions, including but not limited to ‘skills’, ‘social skills’, ‘socio-emotional skills’, ‘interpersonal skills’, and ‘soft skills’ [18]. Moreover, the methods employed for measuring and tracking changes in life skills often lack standardisation and clarity. This is crucial as the research intends to explore the impact of music on young people’s learning development and competencies.

According to the *Life Skills framework* [15, 19], life skills are categorised into three primary domains: cognitive, emotional, and social-relational. The *cognitive domain* encompasses skills such as decision-making, problem-solving, critical thinking, creative thinking, and the ability to learn effectively. *Emotional skills* involve empathy, as well as the capacity to manage and cope with emotions and stress. *Social-relational skills* include effective communication, interpersonal skills, cooperation, conflict resolution, acceptance of differences, concern for others, self-awareness, and self-esteem [20, 21]. Additionally, *‘perceived self-efficacy’* [22] is identified as another crucial transferable life skill, referring to young people’s confidence in their ability to attain specific objectives in their lives, such as learning and playing music, as well as their belief in their capacity to influence events that impact their lives [22].

Previous studies have shown the positive impact of music on young people’s life skills development in several dimensions, especially in the cognitive area [23] and in academic skills [24]. Some other studies have explored young musicians’ skills development in the emotional area [25, 26] and the *social-relation area* [17, 27].

Concerning the impact of music on young people’s life skills, there is more research evidence on *listening to classical music’s* effectiveness on skills [28] than on playing music in an ensemble. Indeed, there is limited research on young people’s participation in classical music ensembles and their life skills development.

Our study investigates the influence of participation in classical music ensembles on the development of life skills among young musicians, adopting their viewpoint. Previous research has indicated that engaging in group music activities, such as ensemble or orchestra participation, can facilitate both music and life skills development [27, 29], encompassing cognitive, emotional, and interpersonal domains [17, 23]. Nevertheless, whether musical group ensemble and training impact life skills is still debated. In the following sections, we discuss the previous literature, the rationale of our research, and the study’s contribution to filling the literature gap.

### Young musician and life skills development

#### Cognitive and academic skills

The literature has explored several collateral benefits of learning music in an ensemble, such as cognitive skills and academic performance. Previous studies showed that learning music can enhance young people’s logical-mathematical skills [16, 30], academic skills [31], spatial-temporal performance [32], verbal memory [33], and visual and auditory memory [34]. According to previous literature [32, 33, 35, 36], learning to play a musical instrument requires several skills, such as sustained attention and concentration, memory, motivation, coordination, responsibility and visual-motor abilities [2].

Music develops young people’s cognitive and academic skills, determines a higher academic achievement score in comparison to students who do not participate in formal music education [37] and can be considered a protective factor for preventing school dropout and antisocial behaviours [38]. Also, it enhances creativity and the growth of academic skills (e.g. literacy and language skills) [35, 39].

In addition, there is relevant evidence about young people’s music educational performance and effectiveness [1]. In this regard, a literature review [37] showed that young people who participate in *formal music education* are more likely to have higher academic achievement scores than students who do not participate in formal music education.

Previous meta-analyses have indicated that music training slightly enhances students’ mathematical [40] and literacy skills [41]. Some studies examining the cognitive and academic benefits of instrumental musical training in childhood and adolescence yielded some promising results, including small but significant effects with short-term training and a slight advantage at baseline in studies with self-selection. Notably, participants who had the opportunity to choose their musical training exhibited slightly higher performance levels. These findings underscore the association between instrumental music education and cognitive skills [24].

Despite the existing evidence, the impact of music training on the aforementioned skills remains contentious, with positive outcomes not consistently replicated [42, 43]. While the studies, as mentioned earlier, have robustly demonstrated a noteworthy correlation between music training and skills development, with some delving into causal inference, it is imperative to acknowledge the divergent findings present within the literature. Some contributions have underscored the absence of a statistically significant association between these variables. Indeed, a meta-analysis [43] showed a negative correlation between study design quality and effect size, suggesting that observed effects of music training may be due to confounding variables, and concluded that there is a lack of well-designed studies on music training to enhance children’s and young people’s intelligence and memory-related skills.

Another meta-analysis from the same authors [44] corroborated these findings, demonstrating a weak and moderately heterogeneous effect of music training programmes on cognitive and academic outcomes. This further underscores the key role of study design on experimental results.

Offering a more nuanced perspective, a meta-analysis [24] revealed a significant positive effect of music training on cognitive and academic skills. However, the authors also underscored the scarcity of well-conducted studies, highlighting the necessity for additional research to establish firmer conclusions regarding the nature and extent of transfer between music training and skills development. Nevertheless, caution is warranted when drawing causal connections between music training and non-musical behaviours [45].

#### Self-confidence and emotional and social-relational skills

*Self-confidence*. Music’s inherent characteristics are ideally suited for facilitating life skills [35], promoting psychological development [46, 47], creating opportunities for self-satisfaction and developing effective interpersonal relationships with other young people [36]. Several studies highlighted the impact of playing and participating in music education programmes on young people’s self-esteem [5, 48]. Moreover, playing and learning music in a group supports music development and self-satisfaction with the impact of musical activities [27, 29].

According to a literature review paper [49], performing publicly in a group (ensemble-orchestra) fosters young people’s self-esteem, self-confidence and enthusiasm [2, 50] and multiple studies have documented that music plays a relevant role in determining personal satisfaction [51, 52] and developing a sense of self-identity [53].

A recent participatory music project involving young people in Scotland [54] has shown increased confidence and self-esteem and improved social skills. This type of programme engages young people from different social classes, especially at the margins of society, reaching them on their terms through music that resonates with their lived experiences [54].

*Emotional and social-relational skills*. We can define emotional skills as the human abilities to recognise, express and regulate emotions in everyday life [55]. Previous research on music and prosocial behaviour has demonstrated that children enhance cooperative and helpful behaviour following joint music-making [25], fostering emotional skills development and promoting positive mental health in children and young people.

A systematic review by Campayo–Muñoz and Cabedo–Mas [50], focused on the role of emotional skill in music education, highlighted the development of several skills, notably teamwork, collaboration, reciprocal respect, tolerance, empathy and a sense of belonging through group music-making. A good ensemble performance can involve teamwork and sharing individual and group goals and expectations [37].

An interesting scoping review research focused on 30 studies and examined the impact of the El Sistema and Sistema-inspired music education on young people who play in music ensembles. It found a positive impact on musical and social-emotional development, with less robust evidence for academic achievement and cognitive skills development [56]. Some pedagogical practices, such as mentoring and peer-to-peer learning, are beneficial to promote teamwork. Previous literature [27, 29] has also highlighted the effectiveness of learning and performing in groups on young people’s self-perception and *interpersonal relationships* while receiving peer mentoring. Furthermore, playing music in a group positively impacts young people’s musical skills development [27].

Being actively involved in cultural and music activities has been shown to increase relationship-building [12, 57], communication, and interpersonal skills [12]. This aspect is particularly true for forms of face-to-face active engagement of young people, especially in a society where many interactions among young people are increasingly developing online. Additionally, several music lessons and training sessions were delivered, especially during and after COVID-19, with a positive impact on the young people’s learning process [58].

Empirical studies reveal that both traditional and online music learning settings significantly enhance students’ skill development. Previous research [59] has demonstrated a positive correlation between music self-efficacy, personal traits like self-esteem and grit, and situational skills such as collaborative playing and practice duration among amateur musicians engaged in online tutorials. Furthermore, as discussed by the authors, online music communities existed before the COVID-19 pandemic, influencing diverse approaches to music learning and teaching. Other contributions [60] emphasise the disparities between online and offline music communities, reflecting on their implications for music education.

Similarly, other researchers [61] highlight distinct participation opportunities in online and offline music teaching and learning communities, underscoring the need for further research in this field. Additionally, the integration of networking technologies in online music teaching, as advocated in the literature [62], offers potential solutions to challenges associated with ensemble playing, such as frustration, as evidenced in primary school orchestra settings. However, in the long -term, we are uncertain about the extent to which the misuse of online platforms can negatively affect the social skills of young people in real life.

Music is an excellent tool for mood regulation [2] that blends regulation strategies [63] and coping skills [64]. Some scholars [65, 66] have also emphasised that music interventions for children and young people with learning needs, such as those on the autism spectrum [65] or with other *emotional needs* [66], can effectively develop several emotional and interpersonal skills. Moreover, music can be an inclusive opportunity to actively involve young people with additional support needs and their families [67].

Young people’s involvement in music groups can contribute to social inclusion and group identity by fostering shared unity and togetherness [40, 68]. Scholars have documented how music participation contributes to the formation of social relationships and promotes a sense of security among participants [69]. Particularly noteworthy is the appreciation among young people for ensemble participation, recognised for its musical and psycho-social benefits [27, 70]. Additionally, long-term musical group interaction positively influences empathy in children and young people [17, 25].

Practising music in groups [54] and planning music events [71] for the local community can tap into the many benefits that community membership promotes [72]. According to other authors [73], music is a social phenomenon that can help to create flourishing communities in which the diversity of individuals is celebrated and the support is shared [74]. The enduring recognition of music’s potential in everyday contexts further underscores its role in community-building efforts.

Furthermore, successful musical activity can enhance an individual’s *sense of social inclusion* [11] and *social cohesion* [75]. Recognising this potential, it becomes imperative to undertake further research and implement novel practices to elucidate how music can be intentionally utilised to augment connectedness and serve as a potent resource for fostering healthy communities [73].

#### Challenges experienced by the young musicians learning in the ensemble

The challenges encountered by young musicians learning in ensemble settings have been explored in the literature [76]. While ensemble playing offers opportunities for shared togetherness and emotional rewards, it also presents various challenges related to physical demands, competition, and social structures [77]. Despite being a valuable avenue for the development of life skills, music learning entails moments of challenge and diverse experiences.

The literature has extensively examined factors contributing to challenges among musicians, including personal health issues [78], socio-emotional difficulties [79], and a range of environmental, professional, and psychological stressors [80]. However, there remains a gap in understanding these challenges comprehensively. For instance, recent works [81] delved into resilience and mental health among classical musicians, underscoring the necessity for further investigation into the relationship between the mental wellbeing of music students and the pressures of their practice routines.

Technical challenges in music practice and performance often coincide with feelings of frustration, as evidenced by research among middle and high school string musicians [82]. Such frustration has become a focal point in discussions surrounding practice behaviours, with guidelines for avoiding it emerging as a prominent theme. Recent studies [83] have further emphasised the detrimental effects of high demands in instrumental practice and performance, mainly when competence and demand are not optimally balanced, leading to feelings of frustration and boredom. Moreover, ensemble playing introduces its own set of challenges. Previous research [79] has noted conflict among ensemble members and feelings of frustration stemming from a lack of engagement and discipline within the group. These challenges underscore the multifaceted nature of musical performance and the importance of addressing both technical and interpersonal aspects in musicians’ training and development.

*Music performance anxiety*. The literature extensively explored music performance anxiety (MPA) as a significant factor in musicians’ experiences. Various definitions have emerged, focusing on the complex interplay of genetic, environmental, experiential, and cognitive factors [84]. MPA is recognised to manifest in multiple dimensions, including cognition, physiological arousal, and behaviour [85, 86].

Extensive research has examined factors associated with music performance anxiety (MPA), including audience presence, setting, timing, and personal variables [87], and multiple treatments for MPA have been proposed [85]. Moreover, MPA has been explored in relation to its underlying cognitive processes [88] and its connection to other anxiety disorders [89]. MPA is a pervasive phenomenon, as demonstrated in an empirical study involving students participating in ensemble rehearsals and concerts [90]. While ensemble rehearsals were associated with lower anxiety levels compared to solo performances and ensemble concerts, the study also revealed relatively high rates of MPA among the participants.

### Rationale and research aims

While there has been a growing research emphasis over the past decade on the impact of playing and learning music in a group [24], previous studies [91] have highlighted positive effects on life skills development. Nonetheless, gaps in the literature persist.

*Firstly*, we have limited research on the development of young people’s life skills through playing in classic music ensembles and engaging in group music training and playing [17, 23, 27, 29].

Whilst the literature extensively covers musical technical skills [92–94] and various types of music ensembles, such as young adult ensembles (e.g., [12]), research specifically targeting classical music ensembles and their influence on adolescent’s life skills remains scarce. Furthermore, the predominant focus of documented music ensemble experiences revolves around school-based programs [33, 35], typically involving children who are already acquainted and share similar social backgrounds.

Secondly, the literature has primarily focused on examining the influence of music on cognitive skills and academic achievement in adolescents, with less attention directed towards emotional and socio-relational skills [24, 91].

Thirdly, there is a notable gap in the literature regarding the comprehensive investigation of adolescents’ primary challenges in participating in classical music ensembles, especially concerning skills development. Nonetheless, previous studies have shed light on the particular difficulties children and young people encounter when coping with specific emotions, such as frustration, anxiety or shame [83, 90].

*Finally*, a significant portion of the existing literature relies on quantitative methods to examine the skills of young musicians. However, there is a noticeable dearth of qualitative research, particularly from the perspective of young musicians themselves, regarding their life skills. Addressing this gap could yield valuable insights for the development of new pedagogical practices and training protocols [91].

In light of these considerations, there is a need for expanded qualitative research concentrating on individuals aged 12 to 18, facilitating a comprehensive exploration of life skills and challenges across various stages of adolescent development. This approach acknowledges the diversity within the adolescent demographic and fosters a deeper understanding of young musicians’ experiences. By incorporating participants from different adolescent age groups, both this research and future studies can capture fresh insights and reflections, providing a holistic perspective on the impact of music training on young people’s lives. Furthermore, this diverse age range enables the identification of both commonalities and unique aspects of adolescents’ journeys in classical music ensemble participation, thereby enhancing the depth and breadth of the study.

Based on the previous literature review, the following research questions are addressed in this paper:

**RQ1.**What music and life skills have young musicians developed through classical music ensembles?
**RQ2.** What are the challenges experienced by young musicians?

This study aimed to explore young musicians’ self-perceived benefits (especially life skills) and challenges while learning music and performing in groups. This aim will be investigated using qualitative data to provide an explorative understanding of the phenomenon under analysis. Qualitative analysis will help understand participants’ direct experience of the complexity of human emotions, opinions, and behaviour. This paper explores adolescents’ perspectives to gain insight into their music experiences and how these experiences influence their daily lives. The findings presented the main results that emerged from the qualitative research phase; nevertheless, understanding the pedagogical context is a prerequisite for contextualising these findings.

## Methodology

### Sample and procedures

The paper focuses on qualitative data collected as part of a broader research project that involved qualitative mixed methods. The primary data collection methods included *semi-structured interviews* (6 interviews with tutors/trainers, 10 with community members such as parents and school teachers, and 15 with young people). Additionally, a *focus group* was conducted with charity trustees. Furthermore, interactive group activities involving 36 children, specifically Year 5 students aged 9–10, were conducted within school settings.

Notably, this paper delves explicitly into the perspectives of 15 adolescents sampled from a group of 12 to 18-year-olds, comprising 7 males and 8 females. These participants were involved in a long-term music-education programme offered by EMAE (Early Music as Education), a charity based in Liverpool (UK), to develop young musicians’ skills.

Specifically, participants had attended the programme for at least one year.

All participants shared the common characteristics of being adolescents. We opted to involve individuals aged 12 to 18 due to their participation in the same ‘orchestra’ and engagement in group training within a classical music ensemble. Furthermore, this cohort’s diverse range of ages often enables younger individuals to learn through peer mentoring and peer-to-peer activities.

We adopted a convenience sampling technique to involve participants in our study. This sampling method is commonly utilised in qualitative research, particularly for demographically and geographically localised samples, thus restricting generalisation to that specific context [95].

Convenience sampling entails selecting participants based on their availability and accessibility to the researcher rather than employing random selection methods. While this approach may not yield a representative sample of the population, it can still offer valuable insights, mainly when the objective is to explore specific phenomena or gather in-depth perspectives from a particular group, as in our case.

Ethical clearance was obtained from the Edge Hill University Ethics Board. Researchers explained the project to the young people involved and provided information sheets for potential participants. If the participant was under 16, consent from their parent/carer was required before approaching the young person. The researcher ran through the information sheet and consent form with the young person and reminded each participant that the project’s involvement was voluntary [96].

### Method

The research employed semi-structured interviews (see S1 Appendix) to explore personal perceptions and opinions on the formative activities delivered by the charity. The interviews lasted, on average, between 45 and 60 minutes and were audio-recorded. They were subsequently transcribed verbatim and anonymised to protect the participants’ identities. Data collection commenced on 9 August 2022 and concluded on 5 December 2022.

### Context & pedagogical framework

The students have been enrolled for several years in a music-education programme run by the charity EMAE. The charity provides a whole range of learning and teaching activities and experiences at no cost, including tutorials, classes, seminars, away days and residentials abroad. Its pedagogical model aims at developing musical as much as interpersonal skills in children and young people. Learning music in an ensemble is at the centre of the pedagogical model: most of the learning activities involving young musicians are planned in groups. Through our study, we explore their impact on participants.

The music -education programme comprises innovative curricula tailored to accommodate the diverse ages and learning needs of the students. The method implemented comprises a series of sequential learning objectives along a three-tier system leading to the acquisition of specific musical skills, aesthetic knowledge and ethical values, ultimately leading to a comprehensive objective: ‘to become a good musician and a good human being’ [97].

### Data analysis

A constructivist epistemological approach to collecting and interpreting qualitative data has been adopted. This approach posits that knowledge is actively constructed by individuals based on their experiences and interactions with the world. A thematic analysis was conducted to explore participants’ opinions regarding their perceptions and to elicit suggestions for supporting people more effectively [98].

Thematic analysis is based on an approach which aims to describe, understand, or interpret participants’ experiences and facilitate reflection on emerging conceptual issues. An interpretative approach was chosen to reconstruct the ‘implicit theories’ of the respondents [99].

A rigorous methodological process was adopted for coding and analysis to avoid the loss of valuable information. All the interviews were transcribed *verbatim* with written permission from the participants. Data gathered from the respondents’ narratives were analysed using NVivo [100]. Two researchers carried out the coding and analysis, but there was also continuous feedback from the whole research team (internal coding) throughout the coding process.

**Fig 1:**
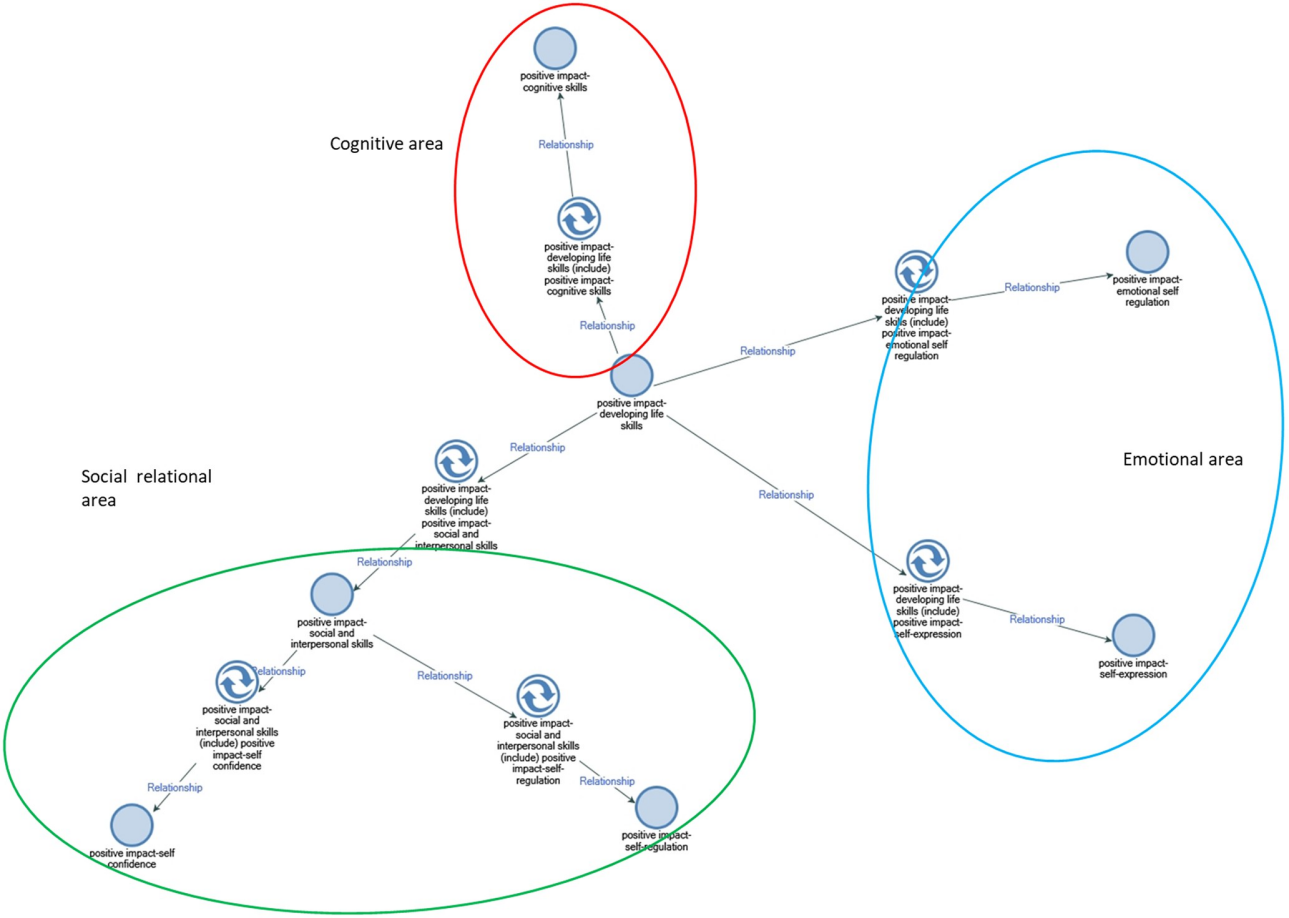
Life skills development.

**Fig 2:**
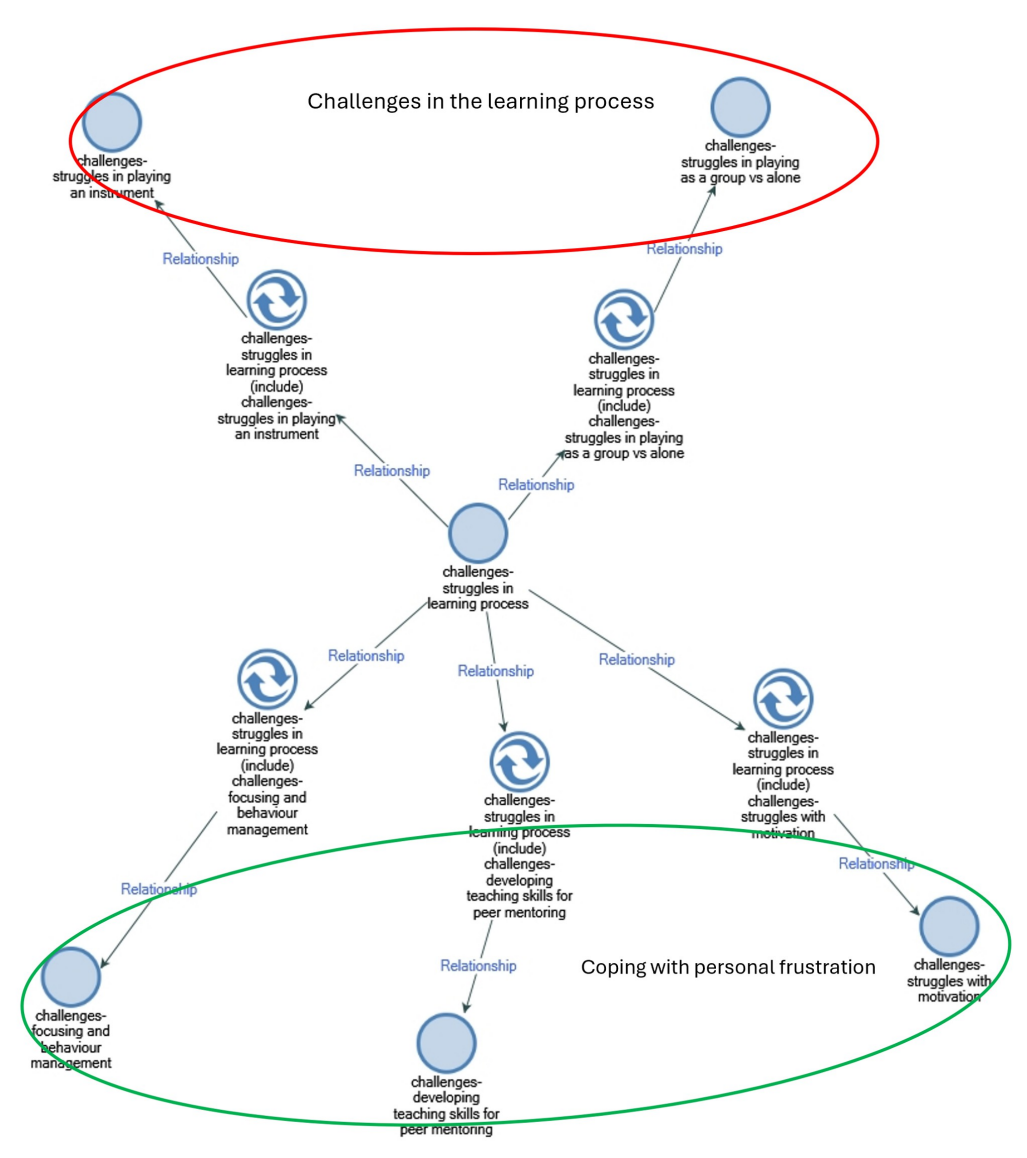
Challenges in the learning process.

The NVivo coding process is not hierarchical but rather inductive. The software provides the “ability to express relationships between codes, concepts, and themes in a range of different ways, and often these cannot be represented in a hierarchical list” [101], 210). A qualitative text analysis elicits themes that resemble and summarise the meaning of participants’ responses (*thematic coding*, also defined as *thematic analysis*). NVivo map (Figs 1 and 2) summarises the interpretation process, which was iterative and progressive. In this respect, the researchers went back to reflect on various conceptual issues and unveil new central aspects [102].

The data was analysed according to the criteria set by Patton [103]. Five quality criteria (i.e., credibility, transferability, dependability, authenticity, confirmability) [104] were applied (see S2 Appendix). Validation was set against these five criteria, with the theme “*saturation*” point [105] reached after 15 participants’ interviews.

As previously explained, the research has gathered multiple viewpoints regarding the subject of inquiry, enabling us to analyse various perspectives on the benefits and challenges associated with young people’s learning in classical music ensembles. In this regard, a triangulation technique was implemented to validate the data through cross-verification from different qualitative sources previously described.

Indeed, methodological triangulation can accurately identify multifaceted aspects by approaching the same phenomenon from different perspectives and using various methods and techniques. [96, 102, 106].

### Main findings

The main units of analysis were *codes* and *nodes*. Codes include ‘nodes’, ‘relationships’, and ‘relationship types’; for the purposes of this analysis, nodes represent thematic categories that are created during the analysis. We also gathered *memos*, representing particularly salient and meaningful quotes from the interview transcriptions.

The analysis generated 473 *codes* focused on *life skills*: 133 codes in the cognitive area, 125 codes in the emotional area, and 215 in the social-relational area.

Furthermore, the analysis generated 58 codes for *challenges*: 47 related to challenges in the learning process and 11 related to managing frustration. Please refer to S3 Appendix for a list of all the nodes. Finally, we gathered 80 memos.

A macro analysis of the data identified 2 key themes that were evident across the corpus of data that emerged from the interviews: (1) *The main benefits of playing and learning in an ensemble*: *music and life skills development*, *and (2) The challenges experienced by the musicians learning in the ensemble*.

### The main benefits of playing and learning in an ensemble: Music and life skills development (RQ1)

The following sections will discuss in more detail the *music and life skills* that emerged during the research, as described by the participants concerning their musical practice and training.

#### Music skills development

The learning programme consists of innovative courses and activities targeting different ages and participants’ learning needs. Participants recognised the positive role played by the learning activities on their musical and *personal development* and expressed appreciation for all the activities in which they participated: weekly practice, musicianship programme, early music string technique, concerts, and the residentials abroad.

The students reflected on how these activities and playing in the ensemble have impacted their personal music skills development, sometimes in unexpected ways, and recognised the quality of the training delivered and what sets this practice apart from other music training. Crucially, this high-quality training has a positive impact on technique development that, in turn, helps the students during their concerts and performances (extract 1) (RQ1).

***Ex 1.** “I would say the most interesting aspect because it is. Developing our technique in a way that we would not explore otherwise. So, our normal music teachers will not teach us these things, and we are getting a very specialised, very high-quality education. And it is helping us to improve our output when we do concerts and things, and it just opens up a new level of expertise and understanding for musicians.”*

*(participant, male).*

#### Life skills development

During the interviews, participants reflected on the courses and activities offered by the charity and how they helped them develop specific life skills (*cognitive*, *emotional*, *social-interpersonal)*. The NVivo Map (Fig 1) summarises the three main areas identified from the thematic analysis of the life skills developed by the participants during the musical learning activities. These areas include cognitive skills, emotional skills (such as emotional self-regulation), and socio-relational skills (including self-confidence and self-regulation).

These findings align with extensive research discussing the positive effects of musical education on emotional intelligence, creativity, and interpersonal relations (see [107] for a review).

As mentioned in the introduction, learning music allows children and young people to develop critical thinking (*cognitive skills dimension*). In our research, participants reflected on how this critical thinking is not limited to music practice but impacts educational and workplace settings. In particular, older participants, most of whom are currently enrolled in higher education, reflected on how the training activities helped them prepare for the workplace and higher education by developing their critical skills (extract 2) and time/self-management (extract 3).

***Ex 2.** “And I think my critical evaluation is better, maybe not the contents of my critical evaluation, but what I look for.”*

*(participant, male).*

***Ex 3.** “I think […] you have to show up on time to your practice. You have to bring your instrument, bring everything. It kind of helps like prepare yourself for like school and all that because going from secondary to sixth form.”*

*(participant, male).*

When reflecting on the impact of the training and group musical activities, participants referred to social-emotional skills like *self-confidence* and reflected on how they may feel more comfortable engaging with others, talking to people, and playing with others because they are in a safe environment that allows them to push their limits at their own pace. Indeed, extract 4 suggests an association between playing in a group and increased confidence in concerts.

***EX 4**. “I think it’s also helped a lot with my confidence, with me playing in front of large audiences with all the concerts and things like that. And as I was saying before, I get quite nervous playing on my own. So, when it’s in the group, it’s just helped a lot with my confidence”*

*(participant, female).*

By playing in an ensemble, students had the opportunity to develop interpersonal skills like *listening* to others and being *aware* of their surroundings. These interpersonal skills (extract 5) are accompanied by individual skills like *resilience* and *motivation* needed to sustain regular intensive musical training (extract 6).

***EX5**. “Guess it’s like maybe a better listener, because you have to, especially with this organisation, this Orchestra, you have to be aware of everything that’s happening. You have to notice what other people are doing.”*

*(participant, male).*

***EX 6.** “So it’s just building all of your character traits music in a way that not many other, erm, walks of life can. It really builds your resilience, your strength, your motivation, in particular your motivation.”*

*(participant, male).*

Optimal music practice does not only rely on *resilience* and *motivation*. Discipline is another key factor for regular and consistent training: participants shared how they learned to manage their negative emotions and anxiety (behavioural and emotional self-regulation) and to follow a precise discipline to maximise their training efforts (extract 7).

***EX 7.** “And the problem with me is that I’m not very disciplined with my practice all the time, so having that discipline just built into the schedule helped a lot”*

*(participant, male).*

In relation to social-relational skills, *connecting with others through music* was a key dimension in the analyses and emerged several times. Young people reflected on how they connected with the broader community and the public and how they created positive experiences through their music. Crucially, positive connections stemming from a shared interest in music and ensemble music practice also impacted other interpersonal connections, and, on the other hand, these developing positive social connections contributed to students’ engagement with and enjoyment of ensemble playing (extract 8).

***Ex 8.** “I think definitely it does help. Like when socialising with people because you already know that you’ve got all play instruments. It’s kind of like you’ve got a common interest and it really kind of like helps to… Kind of like break the ice. I guess you can ask like what instrument do you play or something like that? And then go from there […]”*

*(participant, male).*

Participants enjoyed playing with their peers (extract 9) and “learning from others”, including the charity’s internal and external members.

***EX 9.** “(…)”And then when you’re sharing it with other people who are like minded as you, when you’re all sharing the same interest playing together, it’s as if you can communicate through the music.”*

*(participant, female).*

Furthermore, older students who have left the training programme and started university keep returning to take part in concerts, and friendships started during the musical training have continued at university. Former students have the opportunity to *reconnect with the ensemble* and the youngest members during the concerts (Extract 10). This connection is not only situated in space and time (concerts, rehearsals) but is also extended in time, and musical activities may be used as a starting point to foster the development of broader connections and relationships between the students.

***EX 10.** “I come back for every concert I can. It’s great to engage with the orchestra. It’s very fun.”*

*(participant, male).*

#### Learning activities for supporting skills development

The learning programme allows students to develop skills through regular ensemble practice and mentoring, as well as through international summer residencies and concerts aimed at facilitating community building and interpersonal skills. Another feature of the training programme that facilitates social relational skills and community building is peer mentoring. These peer mentoring activities represent a challenging but meaningful educational opportunity (as discussed in Extract 11 and in the next section).

Students are invited to mentor younger colleagues, helping them develop their music practice and skills. In some instances, they also assist with music training in local primary schools by supporting ad-hoc weekly activities. Participants recognise the importance of their peer mentoring support: students involved in peer mentoring described how it is mutually beneficial for both the mentor and the mentee, allowing the former to reflect on their music practice and the latter to learn from someone they perceive as closer to them than the more experienced tutors (Extracts 11 and 12).

***EX 11.** “And it’s just really nice to help the younger ones how the tutors have helped us and try and pass on what they’ve taught us to the younger ones”*

*(participant, female).*

***EX 12.** “I suppose the biggest benefit is when they do get it, and they do enjoy it, and there’s nothing more fulfilling than seeing a child learn, enjoy music, have fun. That they’re the main benefits of going into primary school and- it’s a highlight of my week. I really do enjoy it”*

*(participant, male).*

In addition to fostering the development of musical skills in both the mentor and the mentee, peer mentoring supported the development of social and emotional skills as well. For example, one of the mentors, a student with ASD needs, was directly involved in training younger students by supporting the tutors in essential musicianship and technique activities (mentoring). When reflecting on these activities, the young musician discussed how this experience helped to develop confidence and satisfaction and contributed further to the sense of community in the charity.

Delivering *concerts* represented a significant learning achievement for the participants, contributing to personal growth, self-awareness, and interpersonal skills. Participants are enthusiastic about the concerts, and they consider them an occasion to put into practice their training and strengthen the social bond in the ensemble. For some students, concerts represent the culminating point of their practice (extract 13).

***EX 13.** “I love the concerts. I think the concerts [are] always such fun cause everyone’s like on edge and like you’ve worked so hard.”*

*(participant, female).*

The mutual connection fostered through music and the development of social skills played a crucial role in promoting community building within the group, another key theme emerging from the interviews. Additionally, international summer residencies were identified as other activities that contributed to *community building* and *fostering connections through music* within the group. Students reflected on the supportive, inclusive, and nurturing atmosphere created by their tutors when discussing these activities (see extract 15). Concerts and international residencies provided students with opportunities to refine their skills, practice, and cultivate social cohesion within the group. Furthermore, for some students, the summer residency served as their inaugural international experience and marked their first time living away from their families.

***EX 14.** “I think you do meet other people and you meet people of like minds and musicians are fairly social in my experience. So you get to widen your social circle.”*

*(participant, male).*

### Challenges experienced by the musicians learning in the ensemble (RQ2)

The second Nvivo Map (Fig 2) illustrates the two main areas that emerged from the thematic analysis of the main challenges shared by the participants regarding their musical training. Students discussed struggles in the learning process, including specific challenges in instrument playing, difficulties in group dynamics compared to individual practice, and challenges related to managing frustration. Furthermore, students expressed frustration with maintaining focus, managing behaviour, staying motivated, and developing the necessary teaching skills for effective peer mentoring.

#### Challenging learning processes

The participants discussed challenges and struggles in their learning process, particularly related to the technical aspects of playing an instrument and adapting to new music styles and techniques. Several of the interviewed students had previously been trained with modern techniques and found it challenging to transition between these methods and traditional approaches (see extract 1).

***EX* 1.** *“But I do find it a little bit difficult to go between modern and baroque*. *So like my current teacher who’s not*, *she’s not a Baroque player*. *She always says that I tend to [unclear] my bow like I can’t*. *I can’t just play like a straight bow anymore because I’m really used to phrasing it in a baroque way*.*”*

*(participant, female).*

Another significant aspect of the learning process concerns the similarities, differences, and overall experiences between playing in a group versus playing alone.

Interestingly, the preference for individual versus group playing was largely subjective, with students expressing their preference for one or the other. Participants reflected on how playing in an ensemble requires musicians to be more receptive to others’ actions, granting them less freedom and choice over what to play (see extract 2). Ensemble playing introduces boundaries and rules in music practice, and students appear to be aware of the importance of these rules and boundaries for optimal ensemble performance. Some students also reflected on how playing alone can be a more stressful experience than playing in a group (see extract 3). Taken together, these two extracts suggest that the boundaries and rules imposed for ensemble music practice may also offer protection and reassurance to musicians who feel nervous when playing alone.

***EX 2.** “When you play with others in an ensemble unless you’re the one that’s taking the ensemble. I think you just have to listen more, and you have to respond and be receptive to what other people are telling you so. I think the way that. You are all playing, and what you’re playing is very different in that way.”*

*(participant, female).*

***EX 3. “**And like I said before, I like playing with erm, lots of different people. And I always get quite nervous when I’m playing on my own.”*

*(participant, female).*

Due to these challenges in the learning process, maintaining the level of discipline necessary to persevere with the training was also identified as one of the challenges described by the students. Students occasionally faced struggles with discipline and motivation, yet they are fully aware of their importance (extract 4).

***EX 4.** “I think the problem is it’s a little bit like you have to have a lot of discipline, like there’s not always motivation. Sometimes you just have to be like I don’t wanna practice… I have to.”*

*(participant, male).*

#### Managing negative emotions

When dealing with these technical challenges, students compare their performance to what the standard should be, leading to feelings of *frustration*, and also compare themselves with the other members of the ensemble, leading to feelings of *not being up to scratch* and *having to catch up with the others* (extracts 5 and 6).

***EX 5.** “And sometimes you get frustrated. I often get frustrated if I’m [unclear] a difficult passage hard and I’m going over it and it’s not working”*

*(participant, male).*

***EX 6*.** “*I really had to really work to catch up to everyone else*. *And that was a lot of*, *like*, *adversity*, *if you will*. *And since it’s a very steep learning curve”*

*(participant, male).*

Social comparison during practice and performance is associated not only with feelings of insecurity about one’s skills, technique, and performance (extracts 7 and 8), but also about the social dynamics of collaborating with others (extract 8).

***EX 7*.** “*What if I am doing everything completely wrong and they’re just not telling me because I don’t know it’s*, *but I guess that’s part of being a student as well*

*(participant, female).*

***Ex 8.** “I was sort of insecure because of my technique and also like because of the social side it was like obviously meeting a lot of new people, I think.”*

*(participant, female).*

Students would feel more insecure during training and rehearsal than during concerts; this may be due to the extensive training and rehearsal they are involved in during the year, especially before crucial performances. Moreover, concerts represent the end goal of many hours of practice and a way to share one’s passion and commitment with the wider public. They may also include a positive emotional component that classes and rehearsals lack. Consequently, concerts and similar exhibitions were usually associated with positive feelings of connectedness and satisfaction (extract 9).

***Ex. 9.** “I think I enjoy the concerts the most because it’s the final thing you’ve been working for ages, so to get there and then complete the concert for the audiences who have sometimes paid to come and watch you”*

*(participant, female).*

As discussed above, the students report being highly focused on their performance and practice and turn to social comparisons to improve and polish their playing technique, not driven by feelings of competition with their colleagues and co-players. Indeed, very few instances of competition emerged from the students’ narration of their training and playing practice.

While students participating in the peer-mentoring scheme reflected on its positive impact, as illustrated in the section on ‘learning activities for supporting skills development,’ some of the learners involved in the scheme also reflected on their challenges with the peer-mentoring programme. For example, they reflected on how it can be challenging for young mentors to share their knowledge effectively and interact with their mentees appropriately to maintain a good rapport, trying to balance assertiveness and kindness while managing their frustration with the teaching practice (extract 10).

***EX 10.** “You can help them get it, you need to try and speak to them in a way even if they are frustrating, then you need to speak to them in a way that you can still maintain a good rapport with them”*

*(Young person, male).*

## Discussion

### Music and skills development

Students’ reflections on their music skills development are consistent with research findings showing the positive effects of *exposure to baroque music* [92, 93] and baroque music practice [94]. Continued and repeated practice is also associated with anatomical changes: neuroimaging findings have shown differences in cellists’ brains when playing contemporary and Baroque styles, suggesting that these various music styles may tap into different cognitive functions [108].

When talking about the impact of their music practice, students referred to musical and life skills. Students’ reflections on critical thinking and evaluation are consistent with previous empirical work [109], showing increased *critical thinking skills* in children undergoing musical training. While this empirical work might not be generalisable to adolescents and young people due to the age range of its sample, it nonetheless drew attention to the central role of adults’ attention and guidance as the key element for children’s improvement. Consistent with this, several students who took part in the present research discussed at length the level and quality of attention they felt they were getting from their tutors.

Moreover, music training may encourage reflection and critical thinking by putting the learners in situations without clear-cut answers [110]. More broadly, findings from the literature indicate that music-making and practice contribute to the development of self-motivation in university students [27] and career professionals [111], suggesting long-term effects of music practice.

In addition to critical thinking skills, participants reflected on how the ensemble training and practice helped them develop *self-confidence and self-efficacy* [59] and on how they developed interpersonal and communication skills through teamwork in an environment with little to no demarcation between playing together as an ensemble and being together as friends or friendly acquaintances. In turn, effective communication and interpersonal skills contributed to community building in the organisation. These outcomes have also been found in previous empirical studies [112] that have highlighted the effectiveness of music on self-confidence in adolescence. These positive effects of teamwork are consistent with findings [91] showing how group music training in third and fourth-grade children (younger than the students who took part in the interviews) was associated with significant increases in sympathy and prosocial behaviour, thus suggesting that group music training could facilitate the development of prosocial skills.

Students also addressed competencies in terms of increased resilience and motivation, consistently with research [113] suggesting that ensemble music training may be associated with neuroplastic changes promoting resilience in children.

While resilience acts at the individual level, *Self-confidence and collective efficacy beliefs* [22] are associated with performance quality for chamber ensembles, helping undertake and overcome musical challenges [114]. Participants also highlighted that playing in an ensemble has improved their self-awareness and listening skills, which are essential characteristics for playing in a musical group but are easily transferable to work and educational settings as well. These positive findings resonate with previous studies suggesting the beneficial effects of music intervention on *social and emotional skills*, *empathy development*, prosocial behaviours, and social and emotional skills [91, 115].

Participants also reflected on how their behavioural and emotional self-regulation developed. *Self-regulation* [116] is an essential skill that involves young people developing the ability to regulate their emotions, thoughts, and behaviours to act positively towards a challenge/task, for example, instilling a sense of discipline needed for continuous and consistent practice as described by our participants. Consistent with what was observed in the present research, previous studies [116] found that children, especially those facing socio-economic hardship, showed more remarkable growth in inhibitory control and self-regulation when involved in musical activities than those who were not.

This type of music program provides opportunities for experiencing happiness and developing self-regulation in preschool children and during the transition to formal schooling [117], as well as fostering emotional regulation skills.

Given the emotions communicated by music, specific early childhood music programs might also promote emotion recognition, which is considered a prerequisite for independent emotion regulation [118], as well as facilitating children’s practice with strategies for regulating emotions via music and movement [117]. Finally, self-regulation is also associated with learning skills and *socially shared self-regulation in group activity* [119], which is in line with students’ reflections on the idea of taking responsibility for one’s playing rather than hiding away.

When talking about the range of activities delivered by the charity, young people mentioned concerts and performances and the connection they allow them to have with the broader community and the public. Active music involvement and participation have been associated with the creation and maintenance of social connections in older adults [120], and general music classes can represent a way to provide middle school students with opportunities for social connections, drawing them together in a music-making community [121] like the one described by our participants. Older students talked about reconnecting with the ensemble: the connections and long-term collaborations between older students and the Orchestra could even take the form of a *collaborative partnership* in which a “deeper, prolonged connection has additional benefits that augment and enrich the musical awareness of members” ([122], p. 34).

### Learning activities for supporting skills development

Participants acknowledged the importance of their *peer mentoring support*; this is in line with previous findings showing how peer support represents an essential component of a student’s support structure in music training and particularly in reducing music performance anxiety [79]. Mentoring can be helpful for building trustworthy relationships among peers and developing self-confidence in both mentors and mentees [20, 29, 123].

Students involved in peer mentoring reflected on the mutually beneficial impact of the activity for both the mentor and the tutee. These positive impressions align with previous research discussing how peer mentoring supports students with problem-solving skills when dealing with music performance issues [124]. *Peer mentoring* in music learning and education is a valuable practice for developing effective student leadership [125]. It offers a practical guide to peer mentoring in music education, enabling music teachers to implement and benefit from this technique with their students. The value of peer mentoring in music training has been discussed in the literature, with evidence suggesting that “working with a mentor or mentee is […] valuable for musicians, irrespective of the actual detail of the knowledge exchanged” [20, 126] and, more generally, that peer tutoring has potential benefits for music education as well as specific limitations (e.g., rejection towards change, reinforcement or transfer of erroneous knowledge) [127].

Delivering *concerts* contributed to personal growth, self-awareness, and interpersonal skills [20, 128].

Community building was a key theme that emerged from the interviews, coherently with previous findings showing an association between music ensembles and a sense of community [129] and strengthening social bonds and social interactions [69]. Summer programmes and international schools are helpful for building skills, knowledge, attitudes and behaviours that promote academic achievement and healthy development [130]. Moreover, in areas with high poverty rates, *summer learning programs* narrow the achievement gap and increase high school graduation rates, college entrance and college completion among low-income and minority youth. These summer learning programs could contribute further to social cohesion in the group, as international trips have been shown to foster social cohesion in graduate students in higher education [131].

### Challenges in learning music

When reflecting on their learning process, students discussed their struggles with the different music styles: Indeed, baroque techniques and instruments differ from modern ones (see [132] as well as [133] for a reflection on the history and evolution of cello vibrato). These two playing styles are so different that it is common for players trained with modern techniques to struggle extensively with baroque techniques [94], as reported by our participants as well.

Another distinction relevant to students’ practice, that emerged in our interviews, is related to playing alone and in a group; students discussed strengths and weaknesses for both settings related to boundaries and limits in practice and the benefits of being around people. Consistent with this, previous research [134] found that chamber music students are inspired by, among others, group discussions and a collaborative atmosphere; vice versa, other studies [135] found that collaboration had a detrimental effect on musician vocalists’ performance in practice. Both situations (individual and ensemble playing) present specific concerns and challenges related to the setting and ongoing interactions in the ensemble. It should also be noted that individual practice is a much more widespread and ‘mainstream’ training choice: “Very little is known about how students learn to practice in a large ensemble” ([136], p. ii) even though ensemble playing presents several positive benefits in terms of musical skills, life skills development, teamwork and community building, among others (e.g. [91]).

In relation to the challenges experienced by students involved in the peer mentoring scheme, young mentors reflected on how it can be hard to share their knowledge effectively and to interact with their mentees appropriately to keep a good rapport. It is thus up to the teacher to build learning scenarios that encourage the appropriate interactions and interdependence between students [127]. These positive interactions with a more knowledgeable peer can then facilitate guided learning and the co-construction of knowledge and skills [91].

As highlighted by our analyses, frustration is a common consequence of challenges in the learning practice. Frustration has been associated with music playing in a survey of practice behaviours among middle and high school string musicians [82]. Specifically, when asked to share practice advice, students seemed sensitive about moments of frustration and shared specific skills to limit it. Frustration has also been associated with very high demands in instrumental practice and performance [83] but has also been shown to decrease as students become more self-regulating in their music practice [137]. Feelings of shame and frustration also emerged in a descriptive study involving advanced violinists’ music practice, all of them pursuing an undergraduate or postgraduate degree in music education [138].

Regarding skill development, ensemble playing can represent a social face-to-face activity carrying intrinsic benefits that go above and beyond its impact on life skills. Scholars, indeed, have reflected on the intrinsic value of the musical experience [139], and on whether the categorisation between intrinsic and extrinsic benefits when discussing the value of music in school is robust and helpful [140]. Another promising approach to the conceptualisation of musical training’s impact and benefits [141] showed that participation in intensive music training is not associated with improvement in executive functioning and self-perception. Nonetheless, it highlighted the importance of the intrinsic value of music education itself.

### Conclusions and recommendations

In a post-COVID era, where young people increasingly socialise with peers and engage in online learning projects (see, for example, [142]), the findings of this research, which analyses ensemble music playing as a social face-to-face activity, underscore the importance of in-person group learning. Such learning environments are crucial not only for acquiring new knowledge but also for developing cognitive, emotional, and interpersonal skills.

The research findings underscore the significance of classical music ensembles in fostering essential music and life skills among young individuals, as well as the multifaceted benefits derived from participation in such ensembles. The research has revealed that students consider participation in classical ensemble music instrumental in enhancing their personal and social competencies. Beyond honing their musical abilities, young participants reported significant advancements in cognitive, emotional, and socio-relational skills through group music engagement. One of the most significant findings is related to the impact of young people’s interpersonal skills on promoting community building, facilitated by playing music together, pedagogical practices aimed at peer support (such as peer mentoring), and the dissemination of a collaborative and social culture. In addition to the benefits observed by young people, the challenges they have faced have the potential to be moments of growth and learning to strengthen self-confidence and resilience [81].

First, the implications drawn from this research suggest that schools, trusts, and music education hubs need to collaborate more closely to provide additional classical music education opportunities for adolescents (aged 12–18), such as “classical music ensemble” programmes. Furthermore, institutions should actively involve children and young people in co-producing advisory groups and participating in classical musical activities and programmes, aiming to design compelling experiences for developing both music and life skills. Given that children and young people are co-constructors of knowledge, it is essential to listen to their voices, particularly in social contexts where resources may be limited.

Given the identified gap in the literature, particularly concerning the target group we have analysed, further research is warranted to explore the impact and effectiveness of classical music programs, with a particular emphasis on ensemble-based initiatives. This is crucial due to the unique characteristics of individual versus group-based practice elucidated in this study. Additionally, there is a need for more qualitative investigations into the experiences and requirements of young musicians participating in classical music ensembles. Furthermore, future studies should examine the disparities in skills development among young people across various music genres, including jazz and rap, compared to classical music.

It would also be important to implement longitudinal studies and evaluate players’ self-perceptions of the impact of music ensembles on their life skills in the long -term and different life dimensions (see, e.g. university or job performance, social life).

Finally, to support adolescents’ learning process through music, we must explore more pedagogical strategies, their impact, and how young people learn to practice in ensembles [136]. Future research must evaluate the impact of new pedagogical models and techniques adopted in ensembles for developing musicians’ music and life skills.

## Supporting information

S1 Appendix (DOCX)

S2 Appendix (DOCX)

S3 Appendix (DOCX)
