# Supplementary material for: Playing music together: Exploring the impact of a classical music ensemble on adolescent’s life skills self-perception
Source: PLoS One. 2024 Jul 11;19(7):e0306326. doi: 10.1371/journal.pone.0306326 (PMC11239010; doi:10.1371/journal.pone.0306326)
Supplement: S1 Appendix — (DOCX) [file pone.0306326.s001.docx]

**Appendix 1 Interview guide**

**Exploring the Impact of Music on Liverpool’s Children, Young People and Families’ psycho-social well-being and skills development**

**Interview**

**Aims:** To explore participants’ perception of musical activities’ impact on young people and children’s psycho-physical and social well-being. To collect personal perceptions on the formative activities delivered by the Charity (e.g. tutorials, classes, seminars, music scores, copies of historical instruments, week-long residency, and a whole range of learning experiences at no cost).

**Introduction**

1. Researcher: Introduce yourself and explain the research

**Opening questions**

1. Could you introduce yourself?
2. When did you start to learn music?
3. How long have you attended/have been attending the activities delivered by the Charity?
4. What do you like about playing an instrument? Some benefits?
5. Did you face any challenges/problems in learning an instrument?
6. How do you feel when you are playing an instrument?
7. What activities do you usually do here? (e.g. tutorials, classes, seminars, music scores, copies of historical instruments, week-long residency, other learning experiences)?
8. What do you like about your experience playing with others?
9. Could you explain the difference between playing with others and alone?
10. What do you think you have learned /are learning during these activities promoted by the Charity?
11. What activity do you like more linked to music?
12. What do you think about your tutors/trainers?
13. What do you think about your peers here? Did you make new friends here? Do you meet them outside the music activities?
14. Do you take part in peer mentoring activities (teaching and supporting children younger than you)?
15. What would you say are the benefits of helping other children?

**Suggestions/ best practices**

1. Suppose that next year, you have the opportunity to plan a new activity for you and your peers related to learning music; what would you like to do? Why?
2. What do you think would be useful in supporting children and young people in learning an instrument? Any suggestions?

**Closure**

1. Do you have any final suggestions and comments?
2. Have we missed anything important in this interview?

**Thank you for your time and collaboration; your suggestions are important, valuable, and interesting.**
