# Supplementary material for: Playing music together: Exploring the impact of a classical music ensemble on adolescent’s life skills self-perception
Source: PLoS One. 2024 Jul 11;19(7):e0306326. doi: 10.1371/journal.pone.0306326 (PMC11239010; doi:10.1371/journal.pone.0306326)
Supplement: S2 Appendix — (DOCX) [file pone.0306326.s002.docx]

| **Appendix 2 Quality criteria**  **Credibility** (internal validity): Member validation involves participants assessing the extent to which they can relate to the researchers' conceptualization of the phenomenon. We solicited feedback from participants on the researcher's interpretation during the interviews, and we shared the research report with them to collect feedback before publication  **Transferability** (external validity): The research team has comprehensively described the methodology, including detailed information on all stages of the research process.  **Dependability:** The research team acknowledges the significance of peer auditing. We collected feedback and suggestions throughout the entire process from researchers and professional experts in the field. This peer review process aids in achieving dependability by clarifying the methodology and findings, thereby enhancing their reliability and trustworthiness  **Authenticity**: The participants have acquired a deeper understanding of the phenomenon explored during the interviews. Each participant reflected on their educational journey, challenges, and acquired skills and knowledge.  **Confirmability**: The research team shared the methodology, coded, and interpreted the information to ensure internal confirmability. The research is also designed to be replicable, ensuring external confirmability. Additionally, the interpretation of data was shared among the team members. |
| --- |
