# Supplementary figures and images for: Playing music together: Exploring the impact of a classical music ensemble on adolescent’s life skills self-perception
Source: PLoS One. 2024 Jul 11;19(7):e0306326. doi: 10.1371/journal.pone.0306326 (PMC11239010; doi:10.1371/journal.pone.0306326)

**Appendix 3 NVivo nodes**

**
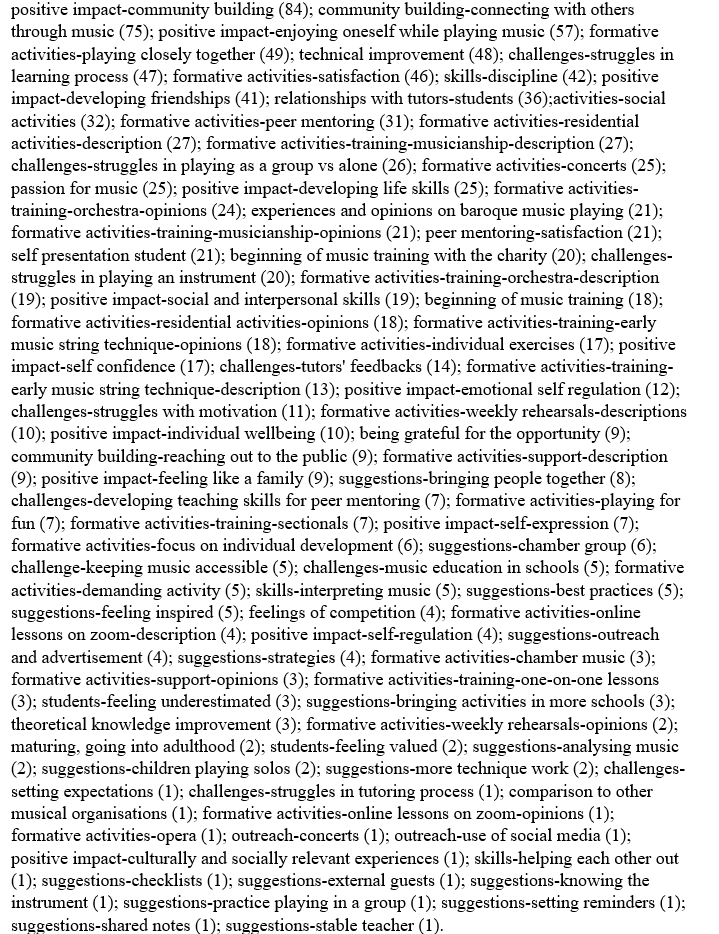
**

Supplement: S3 Appendix — (DOCX) [file pone.0306326.s003.docx]
